# Hospitalisations and outpatient visits for undifferentiated fever attributable to scrub typhus in rural South India: Retrospective cohort and nested case-control study

**DOI:** 10.1371/journal.pntd.0007160

**Published:** 2019-02-25

**Authors:** Carol S. Devamani, John A. J. Prakash, Neal Alexander, Motoi Suzuki, Wolf-Peter Schmidt

**Affiliations:** 1 Department of RUHSA, Christian Medical College, Vellore, India; 2 Department of Clinical Microbiology, Christian Medical College, Vellore, India; 3 MRC Tropical Epidemiology Group, London School of Hygiene and Tropical Medicine, London, United Kingdom; 4 Department of Clinical Medicine, Institute of Tropical Medicine, Nagasaki University, Nagasaki, Japan; 5 Department of Emergency Medicine, Christian Medical College, Vellore, Tamil Nadu, India; 6 Department for Disease Control, London School of Hygiene and Tropical Medicine, London, United Kingdom; Lowell General Hospital, UNITED STATES

## Abstract

**Background:**

The burden of scrub typhus in endemic areas is poorly understood. This study aimed at estimating the proportion of hospitalisations and outpatient visits for undifferentiated fever in the community that may be attributable to scrub typhus.

**Methodology and principal findings:**

The study was a retrospective cohort with a nested case-control study conducted in the South Indian state of Tamil Nadu. We conducted house-to-house screening in 48 villages (42965 people, 11964 households) to identify hospitalised or outpatient cases due to undifferentiated fever during the preceding scrub typhus season. We used scrub typhus IgG to determine past infection. We calculated adjusted odds ratios for the association between IgG positivity and case status. Odds ratios were used to estimate population attributable fractions (PAF) indicating the proportion of hospitalised and outpatient fever cases attributable to scrub typhus. We identified 58 cases of hospitalisation and 236 outpatient treatments. 562 people were enrolled as control group to estimate the background IgG sero-prevalence. IgG prevalence was 20.3% in controls, 26.3% in outpatient cases and 43.1% in hospitalised cases. The PAFs suggested that 29.5% of hospitalisations and 6.1% of outpatient cases may have been due to scrub typhus. In villages with a high IgG prevalence (defined as ≥15% among controls), the corresponding PAFs were 43.4% for hospitalisations and 5.6% for outpatients. The estimated annual incidence of scrub typhus was 0.8/1000 people (0.3/1000 in low, and 1.3/1000 in high prevalence villages). Evidence for recall error suggested that the true incidences may be about twice as high as these figures.

**Conclusions:**

The study suggests scrub typhus as an important cause for febrile hospitalisations in the community. The results confirm the adequacy of empirical treatment for scrub typhus in hospitalised cases with undifferentiated fever. Since scrub typhus may be rare among stable outpatients, the use of empirical treatment remains doubtful in these.

## Author summary

Scrub typhus is an important cause of fever in many Asian countries, including China, India, Vietnam and Japan. It is caused by the intra-cellular bacteria *Orientia tsutsugamushi* and is transmitted to humans by mite larvae (chiggers) which attach to the skin of the host. Scrub typhus is potentially life-threatening but treatable with relatively cheap antibiotics such as doxycycline. The incidence of scrub typhus in the community is not known as most studies were done at hospitals and health posts lacking a clearly defined source-population for patients seeking health care for fever. In this study, the proportion of fever cases due to scrub typhus and the incidence of the infection were estimated by retrospective case identification in a population of 42000 people living in 48 villages in the South Indian state of Tamil Nadu. We found that about 30% to 40% of hospitalisations for undifferentiated fever may be due to scrub typhus. In contrast, scrub typhus accounted for only about 5% of outpatient fever cases. Our findings confirm the need for large population-based cohort studies to better understand the epidemiology of scrub typhus in endemic settings.

## Introduction

Scrub typhus is a febrile illness caused by *Orientia tsutsugamushi*, a bacterial species belonging to the genus *Orientia* (family *Rickettsiaceae*) [1]. The infection is transmitted by the larvae (chiggers) of trombiculid mites which infect mammals as incidental hosts [2]. Scrub typhus occurs over much of tropical and subtropical East Asia, South Asia and South-east Asia. Populous countries like India, China, Bangladesh, Pakistan, Indonesia, Vietnam and Japan are endemic [3]. The disease has recently been identified in Chile [4] and possibly East Africa [5]. One million annual symptomatic infections have been estimated to occur globally but this figure is uncertain [6]. In many endemic areas, scrub typhus accounts for 15% to 30% of febrile illness leading to health care utilisation [7, 8, 9]. Scrub typhus is associated with significant mortality, estimated at 6% to 10% of untreated cases [2, 10]. Mortality in complicated cases remains substantial despite treatment [11]. Mortality for patients with acute respiratory distress syndrome (ARDS), the most common complication, may be up to 25% [11]. Other common complications include meningo-encephalitis, shock and renal failure [9, 11]. Complications may be avoided by early administration of antibiotics such as doxycycline, chloramphenicol or azithromycin [12]. Adverse pregnancy outcomes resulting in stillbirth, prematurity and low birthweight may occur in 40% of pregnant women with scrub typhus [13, 14]. The global burden of scrub typhus has been explored based on cross sectional serological surveys with no clear link to clinical disease [15], or studies relying on passive case detection [16]. The vast majority of studies on scrub typhus are hospital-based, single institution studies, and there is a paucity of population-based epidemiological data. The study was conducted as a pilot study to estimate the proportion of undifferentiated fever cases leading to health care use that may be attributable to scrub typhus, and to obtain an approximate estimate of scrub typhus incidence in the community. The intention was to explore the feasibility of a large prospective cohort study.

## Methods

### Study setting and design

The study was conducted in rural villages in Vellore District, Tamil Nadu (India). The district is characterised by mainly agricultural villages, with rice, sugarcane, sorghum, coconut, pulses and turmeric as major crops. Animal husbandry mainly includes cattle, goats and poultry. The climate is tropical savannah. Monsoon rains occur between June and December. The scrub typhus season approximately lasts from July to February with a peak from November to January. Scrub typhus occurs sporadically between seasons [17]. The study was conceived as a retrospective cohort with a nested case-control study. We conducted house-to-house screening for unspecified febrile illnesses occurring during the preceding scrub typhus season (2017 / 2018) and used serology (scrub typhus IgM and IgG) to determine past infection with scrub typhus. A control group was enrolled through systematic sampling to estimate the background IgM and IgG seroprevalence. The study was conducted between 23^rd^ of March and 30^th^ of June 2018.

### Enrolment of villages

For this pilot study we wished to target villages likely to be endemic for scrub typhus. We used two different approaches to select villages. We first enrolled villages in Vellore district based on hospital admission records at the Christian Medical College Vellore (CMC). Villages were enrolled if at least two scrub typhus cases from that village were admitted to CMC during the three scrub typhus seasons from June 2014 to February 2017, excluding the current season under study (June 2017 to February 2018). We restricted enrolment to villages reachable within one hour from the hospital, resulting in 29 villages. However, because field workers were unable to identify sufficiently many households and cases in these villages, and due to logistical constraints, we then switched to the second approach. We identified a rural area close to Vellore city defined by the Palar River in the south, the limits of Vellore city in the east and the border to Andhra Pradesh in the North, where several villages had met the original eligibility criteria. Assuming this area to be endemic for scrub typhus as a whole we systematically enrolled villages not previously enrolled moving from east to west until the intended number of over 11000 households were screened. This resulted in a further 19 villages.

### Enrolment of cases

Field staff identified the approximate boundaries of a village using satellite images, and then attempt to cover the whole village through house-to-house enquiry. Houses without anyone present were left out, and not revisited. We used the following eligibility criteria for enrolment of a case of undifferentiated febrile illness: 1) aged 12 years or older, 2) hospitalised for febrile illness, or visited an outpatient department, local clinic or pharmacy due to febrile illness at any time between June 2017 and March 2018, 3) cause for febrile illness not known, or described (by respondent or in available health records) as scrub typhus, malaria, dengue, typhoid, meningitis or pneumonia, 4) absence of leg infection, 5) no operation was done at the hospital, 6) no other surgical cause for fever identified from patients memory or available health records, 7) absence of urinary tract infection (only used to exclude cases if urine culture positive), 8) duration of fever of at least 2 days or duration of fever not known, 9) the fever occurred while residing in the study village and health care was sought at a health centre in the district. Hospitalisation was defined as staying at least for one night. All other health care uses were treated as outpatient / pharmacy visit. If a hospitalised case was not present at the time of the interview, we made an appointment with the participants for blood sampling and questionnaire administration. Absent cases meeting the enrolment criteria for outpatients were not revisited due to logistical constraints. Case and control households were geo-referenced using hand-held GPS receivers.

### Enrolment of controls

We enrolled controls through systematic sampling during house-to-house screening, by contacting household members of every 20^th^ house during the walk. Controls were eligible if they had not sought health care due to febrile illness between June 2017 and March 2018, and were living in the study area during that time. Because of concerns that field workers would predominantly enrol older people and females who were deemed more likely to be present, we used a stratified enrolment procedure, using four strata: females ≥50 years old, females <50 years old, males ≥50 years old, males <50 years old. Field workers enrolled controls in blocks of four, with each stratum being represented once. The aim of this procedure was to obtain a reasonably age and sex balanced control group without requiring a formal sampling frame. Controls were asked to give a blood sample and were asked whether they had had any high grade fever not leading to health care use between June 2017 and March 2018.

### Laboratory analysis

**Fig 1:**
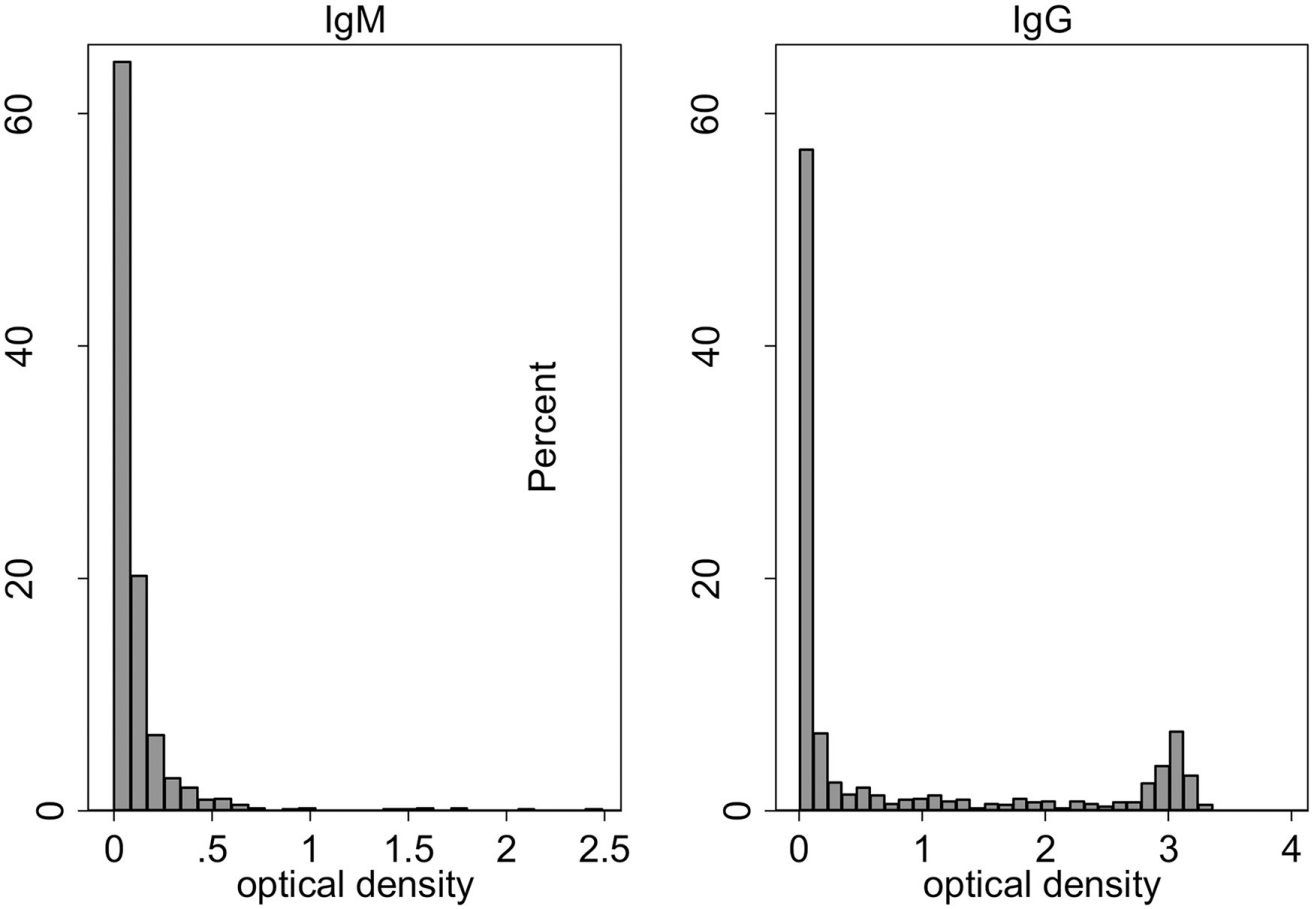
Optical densities for cases and controls, left panel IgM, right panel IgG.

After collection, blood samples were brought to CMC. Serum was separated from blood cells, divided into 3 aliquots and stored at -70°C until testing. We used enzyme-linked immunosorbent assays (ELISA) to detect IgG and IgM antibodies to scrub typhus (Scrub Typhus Detect, InBios International, Inc., Seattle, WA, USA) following the manufacturer’s specifications. This ELISA uses Karp, Kato, Gilliam and TA716 recombinant proteins of the 56-kD outer membrane protein. Commercially available ELISA IgM assays, such as the ones used in this study, have been shown to have a sensitivity and specificity of over 90% in a study from Thailand [18], and 80% sensitivity and 96% specificity in a study from South India [19]. The sensitivity and specificity of IgG ELISA to detect past infection remains to be determined. The distributions of the optical densities for IgG and IgM are shown in Fig 1. Given the pronounced bimodal shape of the distribution for IgG, we chose the apparent midpoint of 1.5 as the OD cut-off point for this study. For IgM, the choice for the cut-off point was made difficult by the unusual application of the test, which has been developed to diagnose acute infection, not past infection. IgM is known to wane during the months following scrub typhus infection, although precise data on the timing of the decline have not been published. The OD cut-off point of 1.0 currently used at CMC for acute scrub typhus infection would have been inappropriately conservative. To avoid lack of specificity, we determined a suitable OD cut-off post-hoc so that IgM prevalence in controls was below 5%. Given this ad hoc approach to determine a cut-off we decided to use the IgM results only in the sensitivity analysis, not for the primary analysis.

### Sample size and statistical analysis

The sample size calculation was based on a preparatory study done by medical interns (CMC, Vellore) to test the questionnaire in three villages not included in the present study. In this study, 11 hospitalisations of unspecified febrile illness over the approximate length of a scrub typhus season were identified with a denominator of 2271 people (cumulative incidence of 4.8 per 1000 people aged 12 years or older). We assumed treatment as outpatient or at a local pharmacy to be 3 times more common (about 15 cases per 1000). We anticipated a population attributable fraction (PAF) of scrub typhus of 25% of hospitalisations, i.e. an incidence of hospitalisation due to scrub typhus of 1.2/1000 people and aimed at identifying at least 25 cases of scrub typhus hospitalisation (out of 100 total cases of hospitalisation). Assuming 30% of households refusing to participate, 30% of cases not being remembered, and a household size of 3.6 (aged 12 years or older), resulted in 11800 households to be screened for active case detection.

The 95% confidence interval for the sero-prevalence of scrub typhus IgG among controls was calculated using linearized standard errors (*svy*: *proportion* command in STATA 12.0, Statacorp). Continuous variables were compared across groups using the t-test. Differences in IgG sero-prevalence by sex were tested using the chi-square test. Adjusted differences in IgG sero-prevalence by sex were calculated using binomial regression (identity link function). The intra-class correlation coefficient for village clustering of IgG prevalence was calculated using the *loneway* command in STATA 12.0. The spatial correlogram for IgG prevalence in controls was estimated using the *ncf* package in R (R project). We used 100m increments. P values for Moran’s I were estimated based on 500 resampling rounds. The sero-prevalence of IgM and IgG was compared between cases and controls using logistic regression. Odds ratios were adjusted for age and sex. We used robust standard errors to estimate 95% confidence intervals accounting for the village-level sampling approach. Interactions between sex and IgG sero-positivity, and age and IgG sero-positivity were explored using likelihood ratio tests. The PAF is commonly defined as the proportion of cases (here: hospitalisation or outpatient / pharmacy treatment) that could be prevented if an exposure (here: scrub typhus infection) were removed from the population. Missing data were ignored in the analysis. We calculated the PAF based on the following formula [20]:

$$PAF\;=\;p\;\times \;\frac{AOR-1}{AOR}$$

where *p* is the prevalence of scrub typhus IgG or IgM positivity in cases, and AOR the age/sex adjusted odds ratio of IgG or IgM sero-prevalence between cases and controls. Note that the formula is only meaningful for odds ratios of 1 or greater. The incidence of scrub typhus was estimated using the number of cases as nominator and the number of screened individuals as denominator, multiplied by the PAF.

### Ethics

The study was approved by CMC’s Institutional Review Board and LSHTM’s Research Ethics Committee. Written consent was obtained from all adult participants. Written or verbal assent was obtained from minors, alongside written consent from their parents/guardians.

## Results

Between 23^rd^ of March and 30^th^ of June 2018, we screened 42965 people living in 11964 households from 48 villages. The mean number of people screened per village was 895 (range 150–2320). We identified 60 hospitalisations and 264 outpatient treatments reported as having occurred between 1^st^ of June 2017 and 31^st^ of March 2018. Among these, samples were obtained from 58 of 60 cases with hospitalisation (97%) and 236 of 264 cases with outpatient treatment (89%) for undifferentiated fever. Two hospitalised cases (3%) and 28 outpatient cases (11%) were unavailable for blood sampling and could not be contacted for a revisit. Data on fever duration was missing in 5 outpatient cases (2%), data on duration of hospital stay was missing in one hospital case. Of the 236 outpatient cases, 15 reported having visited a local pharmacy for diagnosis and treatment rather than a health centre. Only 19 out of 294 fever cases remembered a clinical diagnosis or were able to show clinical records mentioning a diagnosis. Diagnoses made were typhoid (n = 16), dengue (n = 2) and TB meningitis (n = 1). A total of 562 people were enrolled as control group through systematic sampling. Of these, 4 (0.7%) reported a febrile illness having occurred between 1^st^ of June 2017 and 31^st^ of March 2018, for which they did not seek treatment. The mean number of controls per village was 11.7 (range 1–33). Basic characteristics of cases and controls are shown in Table 1. Age was well balanced across groups. The proportion of females among study participants was similar in hospitalised cases and controls, but higher for cases reporting an outpatient visit. Fever duration was on average 3.8 days longer for hospital cases compared to outpatients (95% CI of the difference 2.3 to 5.4).

**Table 1:** Characteristics of cases and controls.

	Hospital cases	Outpatient / pharmacy cases	Controls
Total (n)	58	236	562
Age in years (mean, range)	44.8 (13–80)	44.9 (12–85)	45.0 (13–88)
Female sex, %	53.4	79.7	58.9
Duration of fever in days (mean, range)*	8.7 (2–30)	4.9 (2–30)	NA
Duration of hospital stay in days (mean, range)§	5.4 (1–30)	NA	NA

*data missing in 5 outpatients,

^§^data missing in one hospital case

### IgG sero-prevalence among controls

**Fig 2:**
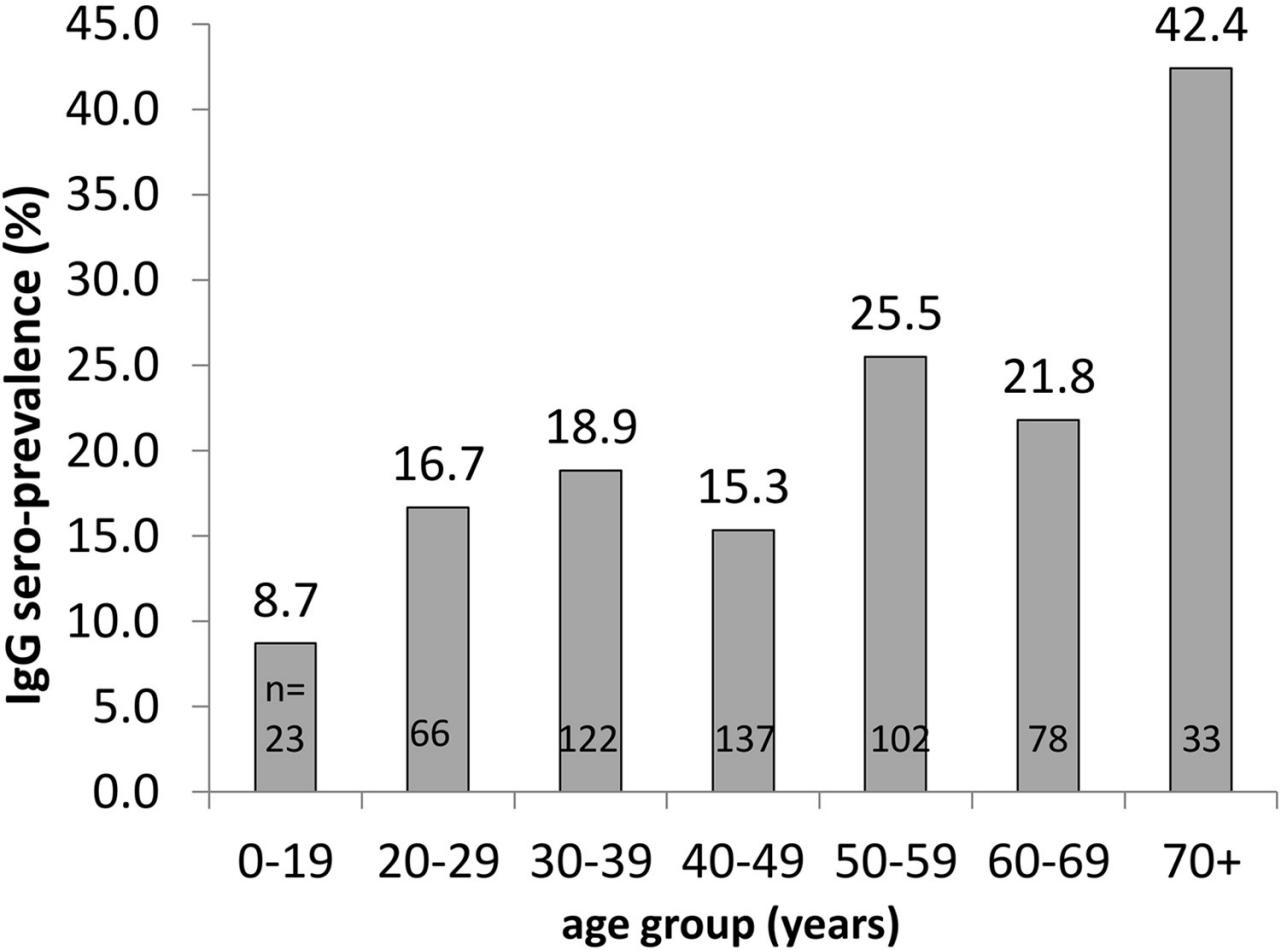
IgG sero-prevalence by age among controls.

**Fig 3:**
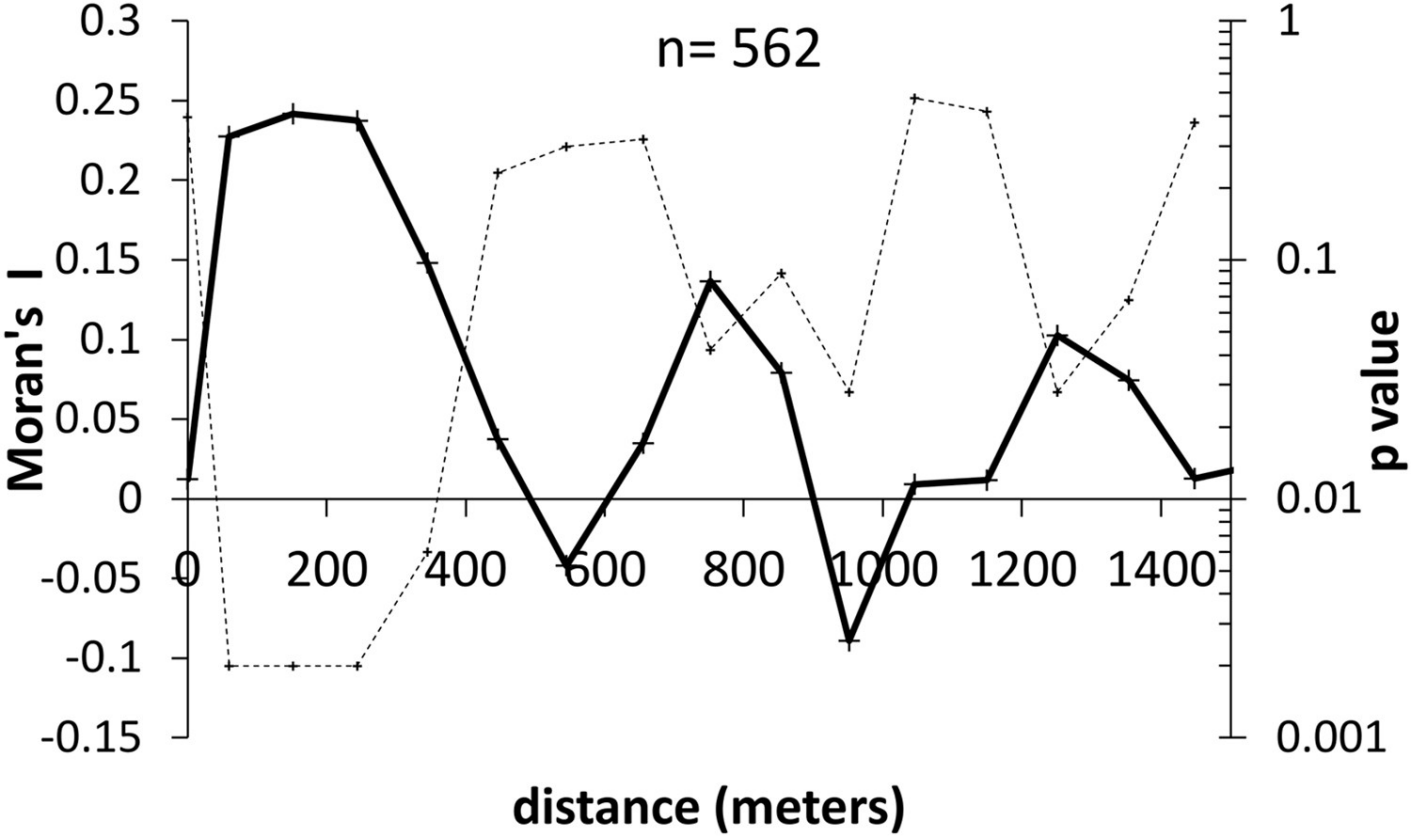
Spatial correlogram of IgG sero-prevalence among controls. Solid line Moran’s I values. Dotted line corresponding p value.

The overall IgG sero-prevalence among controls was 20.3% (95% CI 14.0% to 26.5%). Among controls there was a near-linear relationship between age and IgG sero-prevalence (Fig 2). Female controls were younger than male controls (43.9 years vs 46.7 years, p = 0.03). Sero-prevalence was slightly higher among control females than males (21.5% vs 18.6%, p = 0.411). This difference of 2.9% increased to 3.6% after adjusting for age (binomial regression, identity link). The IgG sero-prevalence among controls varied between villages. Seventeen villages had 0% sero-prevalence while 10 villages had a sero-prevalence of 40% or higher. The variation was higher than expected by chance, indicated by an intra-class correlation coefficient of 0.16 (95% CI 0.07 to 0.25). The spatial correlogram (Fig 3) suggests spatial auto-correlation of IgG sero-positivity largely occurring within 300m.

### IgG sero-prevalence among cases and controls and population attributable fractions

The prevalence of scrub typhus IgG sero-positivity was 20% in controls, 26% in outpatients and 43% in hospital cases. Among IgG sero-positive individuals (OD≥ 1.5) the mean OD was 2.8 for controls (range 1.6–3.4, SD 0.5), 2.7 for outpatients (range 1.5–3.2, SD 0.5) and 2.8 for hospital cases (range 1.6–3.2, SD 0.5). Similar to the total study population (Fig 1), all three groups displayed a pronounced bimodal pattern of OD values (S1 Fig). The proportion of cases attributable to scrub typhus (calculated based on IgG) was generally much larger for hospitalised cases than for outpatient / pharmacy cases (Table 2). The PAFs for hospitalisation exceeded 40% in women, participants aged 36 to 50 years and those living in villages with an IgG sero-prevalence of 15% or higher. The PAF for hospitalisation was only about 10% in men and negligible for participants aged 35 years or younger. In logistic regression, the test for interaction between sex and IgG positivity as predictors of hospitalisation showed a p value of p = 0.046, indicating support for women having higher PAFs for scrub typhus than men. The p value for the test for interaction between age 35 years or under/above 35 and IgG positivity was p = 0.024, suggesting hospitalisations in older ages may be more likely to be due to scrub typhus than in younger ages. There were trends for IgG positive hospital cases having a shorter average duration of fever (8.0 vs 9.3 days, p = 0.41) and a shorter duration of hospital stay (4.5 vs 6.1 days, p = 0.35) than IgG negative hospital cases. However, due to small case numbers and large variations in fever duration and hospital stay, the p values indicated low statistical support for these differences.

**Table 2:** Scrub typhus IgG sero-prevalence in hospitalised cases and controls, and estimated population attributable fractions.

	N	IgG+ (%)	OR	95%CI	AOR*	95%CI	PAF
**Overall**							
Control	562	20.3%	Ref	-	ref	-	-
Outpatient	236	26.3%	1.4	1.0 to 2.0	1.3	0.9 to 1.9	6.1%
Hospitalised	58	43.1%	3.0	1.6 to 5.5	3.2	1.7 to 6.1	29.5%
**By gender**							
Females							
Control	331	21.5%	Ref	-	ref	-	-
Outpatient	188	29.8%	1.6	1.0 to 2.3	1.5	1.0 to 2.3	10.3%
Hospitalised	31	58.1%	5.1	2.3 to 11.0	4.9	2.3 to 10.7	46.3%
Males							
Control	231	18.6%	Ref	-	ref	-	-
Outpatient	48	12.5%	0.6	0.2 to 1.6	0.7	0.3 to 1.6	NA
Hospitalised	27	25.9%	1.5	0.5 to 5.0	1.7	0.5 to 5.5	10.5%
**By age**							
Age ≤35 years							
Control	153	17.0%	Ref	-	ref	-	-
Outpatient	76	17.1%	1.0	0.6 to 1.6	0.9	0.4 to 1.8	NA
Hospitalised	19	15.8%	0.9	0.3 to 3.1	1.1	0.3 to 3.6	0.8%
Age >35 to 50 years							
Control	222	16.7%	Ref	-	ref	-	-
Outpatient	66	31.8%	2.3	1.2 to 4.4	2.5	1.2 to 5.1	19.0%
Hospitalised	16	56.3%	6.4	1.8 to 22.8	6.4	1.8 to 22.5	47.4%
Age >50 years							
Control	187	27.3%	Ref	-	ref	-	-
Outpatient	94	29.8%	1.1	0.6 to 2.0	1.0	0.5 to 1.8	0.0%
Hospitalised	23	56.5%	3.5	1.8 to 6.8	3.4	1.7 to 6.6	39.8%
**By village level IgG prevalence**							
Low risk areas (IgG prevalence in controls <15%)							
Control	254	4.7%	Ref	-	ref	-	-
Outpatient	84	6.0%	1.3	0.4 to 4.1	1.2	0.4 to 4.0	1.0%
Hospitalised	29	24.1%	6.4	2.5 to 16.7	7.1	2.6 to 19.1	20.7%
High risk areas (IgG prevalence in controls ≥15%)							
Control	308	33.1%	Ref	-	ref	-	-
Outpatient	152	37.5%	1.2	0.9 to 1.6	1.2	0.8 to 1.7	5.6%
Hospitalised	29	62.1%	3.3	1.5 to 7.3	3.3	1.3 to 8.3	43.4%

*logistic regression adjusted for sex and age (except in analyses stratified by one of those variables)

### Incidence of scrub typhus

The cumulative incidence of hospitalisation and outpatient / pharmacy treatment over the 2017 / 2018 season is shown in Table 3. Because of the high potential for under-reporting especially of outpatient / pharmacy visits, these data should be interpreted with caution. However, they suggest a higher incidence of scrub typhus in villages with an IgG sero-prevalence of 15% or higher. The higher crude incidence of hospital admission in high prevalence villages (1.7 vs 1.2 per 1000) may be explained by scrub typhus leading to an excess of hospitalisations compared to low prevalence villages. The large difference in outpatient / pharmacy visits between high and low prevalence villages (9.5 vs 3.7 per 1000) is unlikely to be due to scrub typhus given the small PAFs for scrub typhus in outpatients (Table 3).

**Table 3:** Incidence of scrub typhus.

	People	Cases			Incidence / 1000 people			Incidence scrub typhus / 1000 people*		
		Hospital	Outpatient / pharmacy	Total cases	Hospital	Outpatient / pharmacy	Total	Hospital	Outpatient / pharmacy	Total
**Total**	42965	60	264	324	1.4	6.1	7.5	0.4	0.4	0.8
**Village-level IgG prevalence <15%**	24844	29	91	120	1.2	3.7	4.8	0.2	0.0	0.3
**Village-level IgG prevalence ≥15%**	18121	31	173	204	1.7	9.5	11.3	0.7	0.5	1.3

*using a PAF of 6.1%/29.5% for the total incidence (outpatients/hospital), 1.0%/20.7% for low prevalence villages and 5.6%/43.4% for high prevalence villages

### Sensitivity analysis

Focussing on the overall, unstratified analysis (Table 2, top), we tested different OD values to explore how different cut-offs for IgG OD affected the PAF estimates. As shown in Table 4, the IgG sero-prevalence values and the PAFs for outpatient/pharmacy and hospital cases were fairly robust to varying the OD cut-off between 1.2 and 2.0. By contrast, changing the OD cut-off for IgM substantially affected the IgM sero-prevalence estimates. An OD cut-off of 0.2 maximised the difference between hospitalised cases and controls, probably at the cost of low specificity. We used an OD of 0.4 as the default cut-off for the IgM to explore the robustness of the PAFs calculated in Table 2 for different strata to using IgM instead of IgG prevalence. As shown in Table 5, the PAFs are lower compared to the PAFs estimated based on IgG, but reflect the higher risk in women, in those aged 35 years or older and those living in high prevalence villages.

**Table 4:** Sensitivity analysis.

**Scrub typhus IgG**						
Optical Density cut-off for IgG ELISA	1.2	1.4	1.5 (default)	1.8	2.0	2.5
Sero-prevalence						
Controls	22.4%	20.3%	20.3%	19.2%	18.0%	15.7
Outpatient	27.1%	26.7%	26.3%	22.9%	21.6%	20.8
Hospitalised	43.1%	43.1%	43.1%	41.4%	37.9%	31.3
Adjusted odds ratio*						
Controls	Ref	ref	Ref	ref	ref	ref
Outpatient	1.2	1.3	1.3	1.2	1.2	1.3
Hospitalised	2.8	3.2	3.2	3.1	3.0	2.6
Population attributable fraction						
Outpatient	4.3%	6.6%	6.1%	3.2%	3.1%	4.8%
Hospitalised	27.8%	29.5%	29.5%	28.2%	25.1%	19.3%
**Scrub typhus IgM**						
Optical Density cut-off for IgM ELISA	0.2	0.3	0.4 (default)	0.5	0.8	1.0
Sero-prevalence						
Controls	12.1%	6.2%	3.9%	2.3%	0.9%	0.5%
Outpatient	9.3%	4.7%	3.0%	2.1%	1.3%	1.3%
Hospitalised	34.5%	24.1%	19.0%	15.5%	5.2%	3.5%
Adjusted odds ratio*						
Controls	Ref	ref	Ref	ref	ref	ref
Outpatient	0.7	0.7	0.7	0.8	1.3	2.4
Hospitalised	4.0	5.0	6.0	8.0	6.0	6.6
Population attributable fraction						
Outpatient	NA	NA	NA	NA	0.3%	0.8%
Hospitalised	25.8%	19.3%	15.8%	13.6%	4.3%	3.0%

*logistic regression adjusted for sex and age

**Table 5:** Scrub typhus IgM sero-prevalence in hospitalised cases and controls, and estimated population attributable fractions (OD cut-off of 0.4).

	N	IgM+ (%)	AOR*	95%CI	PAF
**Overall**					
Control	562	3.9%	Ref	-	-
Outpatient	236	3.0%	0.7	0.3 to 1.4	NA
Hospitalised	58	19.0%	6.0	3.2 to 11.2	15.8%
**By gender**					
Females					
Control	331	4.5%	Ref	-	-
Outpatient	188	3.7%	0.8	0.4 to 1.7	NA
Hospitalised	31	22.6%	6.1	2.4 to 15.6	18.9%
Males					
Control	231	3.0%	Ref	-	-
Outpatient	48	0.0%	-	-	NA
Hospitalised	27	14.8%	5.9	1.4 to 23.9	12.3%
**By age**					
Age ≤35 years					
Control	153	3.3%	Ref	-	-
Outpatient	76	2.6%	0.6	0.1 to 3.3	NA
Hospitalised	19	10.5%	4.3	0.7 to 26.1	8.1%
Age >35 to 50 years					
Control	222	5.4%	Ref	-	-
Outpatient	66	1.5%	0.3	0.1 to 1.2	NA
Hospitalised	16	25.0%	6.0	1.6 to 21.8	20.8%
Age >50 years					
Control	187	2.7%	Ref	-	-
Outpatient	94	4.3%	1.5	0.6 to 3.6	1.4%
Hospitalised	23	21.7%	10.2	2.6 to 39.4	19.6%
**by village level IgG prevalence**					
Low risk areas (IgG prevalence in controls <15%)					
Control	254	2.0%	Ref	-	-
Outpatient	84	2.4%	0.9	0.2 to 3.8	NA
Hospitalised	29	6.9%	4.4	0.6 to 33.0	5.3%
High risk areas (IgG prevalence in controls ≥15%)					
Control	308	5.5%	Ref	-	-
Outpatient	152	3.3%	0.6	0.2 to 1.4	NA
Hospitalised	29	31.0%	7.6	4.1 to 14.0	27.0%

*logistic regression adjusted for sex and age (age / sex removed in respective analyses stratified by age and sex)

## Discussion

The study suggests that scrub typhus accounts for a substantial proportion of hospitalisations due to undifferentiated fever in this South Indian rural setting. By contrast, scrub typhus may not be a common cause of fever among cases managed as outpatients, even in areas with a high IgG sero-prevalence. The overall estimate of about 30% of undifferentiated fever hospitalisations being attributable to scrub typhus may be realistic: first, we found a strong relationship between village-level sero-prevalence and PAF estimates, which lends some support to the assumption that scrub typhus may indeed be the cause of many of these hospitalisations. Second, the proportion is similar to the proportion of fever cases admitted to CMC that are due to scrub typhus (35.9%)[9], even though this comparison is made difficult by CMC being a tertiary care centre receiving patients from throughout the region, often with complicated infection. Third, the results confirm the observation by local medical staff that the proportion of scrub typhus cases requiring admission appears to be high compared to other undifferentiated fevers such as dengue or respiratory viral infections. The results further suggested a higher PAF of hospitalisations due to scrub typhus in people aged 35 and older, and in women. Due to small case numbers, the stratified analyses need to be treated with caution.

Given the substantial PAFs of hospitalisations due to scrub typhus, the findings support the presumptive treatment with doxycycline of hospitalised fever cases. The scope for presumptive treatment of stable fever patients managed as outpatients seems more doubtful, as only a small proportion of these cases may be due to scrub typhus, even in settings where the sero-prevalence exceeds 15%. This confirms the role for rapid diagnostic tests or risk scoring based on clinical findings [21] particularly in outpatients.

Our estimate of the annual scrub typhus incidence in the study communities (between 0.3/1000 in low prevalence areas and 1.3/1000 in high prevalence areas) is difficult to compare with other studies as these (being hospital-based) usually lacked a clearly defined denominator. A Malaysian study from the 1970s which relied on passive case finding but appears to include many mild cases while lacking a well-defined denominator, suggested an annual incidence of 12 per 1000. This is substantially higher than our estimate, even when accounting for under-reporting in our study. It could be due to the particularly high risk in the selected study area (communities working at palm oil plantations).

The study is limited primarily by the indirect way of estimating case numbers which, not being a prospective design, could not be based on individual case confirmation. Because of the high background IgG positivity in controls, the PAF approach does not allow classifying an individual as a scrub typhus case or not. Estimation of case numbers only refers to the population under study as a whole. The concept of the PAF has been developed mainly for non-infectious diseases to determine the proportion of cases that could be prevented if a given risk factor is completely eliminated from the population. The PAF is not ideally suited to study the aetiology of common conditions of presumed infectious origin such as diarrhoea, respiratory illness or fever, although it is often used in this context, (e.g. Kotloff and colleagues [22] and Smith and colleagues [23]). The PAFs calculated here assume that pre-existing antibodies do not affect the risk of subsequent scrub typhus infection. They would overestimate the proportion of cases due to scrub typhus if IgG was protective. While it is known that one individual can be infected with scrub typhus repeatedly even within two seasons [24], partial protection is a possibility. On the other hand, cases with positive IgG antibodies could represent a subgroup of the population at particular risk of scrub typhus. In this subgroup, more cases could be due to scrub typhus in the current season than suggested by the PAF, which does not account for the possibility of repeat infection. In this scenario, the PAF may underestimate the proportion of cases due to scrub typhus.

The study is further limited by recall error, the possibility of cross reactivity (see below), uncertainty in the choice of cut-off points for the diagnostic tests, and the non-random enrolment of villages into the study. We found evidence that recall error reduced the reported incidence of hospitalisation and outpatient care use. The pilot study suggested a three times higher incidence of hospitalisation due to undifferentiated fever, which may have been due to medical interns being more able to elicit a history of febrile illnesses occurring several months ago than the nurses employed for the main study. It could therefore be argued that the true incidence of scrub typhus in the community may be twice or three times as high as estimated in this study. Following this logic, plausible figures for the annual incidence could be 1.5 to 2.5 per 1000 people overall, and between 2.5 and 4 per 1000 people in high prevalence villages. It seems unlikely that the true incidence of scrub typhus in this setting will be much higher than that. Most respondents were able to remember past episodes of health care use after engaging them in a conversation to build trust and explain the study purpose, but nurses found it difficult to do this while screening a large number of households per day. The potential for recall error to affect the PAF estimates, which do not rely on all cases in the community being identified, is smaller than for incidence estimates. Nevertheless, it is possible that the cases of hospitalisation and outpatient treatment identified in this study are not a random subset of the total cases. Fever duration and hospital stay were somewhat shorter for IgG positive compared to IgG negative cases (p-values indicated that these differences could have been by chance). It seems unlikely for recall error to be strongly associated with IgG positivity, especially not in a way overestimating the PAF for hospitalisations due to scrub typhus.

ELISA tests for scrub typhus antibodies are thought to cross-react with a range of infections such as leptospirosis, tick-borne spotted fever, murine typhus and others. Including a control group largely controls for antibodies circulating in the general population independent of febrile illness but may be subject to misclassification of cases of febrile illness. For example, if a substantial proportion of febrile illness cases enrolled in this study were due to leptospirosis or spotted fever, and cross-reactivity was substantial, then the PAFs calculated here would overestimate the proportion of fevers due to scrub typhus. Leptospirosis and spotted fevers are probably too rare in the study area to affect the findings [9]. Murine typhus and spotted fever group rickettsiosis antibodies may be particularly prone to cross-react with scrub typhus antigens as these are caused by related organisms. A study from an urban setting in Laos jointly examined the prevalence of positive scrub typhus and murine typhus IgG antibodies (based on ELISA tests) in the general population demonstrating different geographic risk factors for the two infections [25]. In this study of 2002 people, 314 were found to be positive only for scrub typhus antibodies, 360 positive only for murine typhus antibodies, while 80 were positive for both antibodies. The expected number of dual positives assuming independence between the two infections would be 86 cases—very similar to the observed 80. If cross reactivity were substantial, one would expect a higher proportion of participants to be positive for both infections. This finding suggests that cross-reactivity, while of clinical relevance especially in acute cases, may be of lesser importance in serological surveys using IgG. However, co-infection and cross-reactivity pattern may differ between Laos and India. Large prospective studies in India are clearly needed to further explore the issue of cross-reactivity.

In the absence of a generally agreed method to determine suitable cut-off points for OD values from ELISA tests, the cut-off points in this study were chosen based on the data collected. However, the sensitivity analysis of different IgG OD cut-off points broadly confirmed the main findings. The results were also broadly confirmed when using IgM even at the chosen cut-off point of an OD of 0.4. The PAFs for using IgM were lower than for IgG probably because IgM declines faster than IgG. When using an IgM OD cut-off of 1.0 currently applied at CMC to confirm active cases, then only 0.5% of controls and 3.5% of hospitalised cases would have been IgM positive (Table 4). The low IgM prevalence in cases and controls when using a conventional cut-off in this study suggest a rapid decline within a few weeks or months after infection. If a blood sample can be collected within weeks of a febrile illness, then IgM ELISAs should be a suitable tool for retrospective case identification in large cohort studies. The longevity of IgG and IgM antibodies is still under debate [26, 27]. The simplest explanation for the strong increase of IgG prevalence with age may be that IgG antibodies remain positive for many years after infection. This is supported by a similar mean, standard deviation and range of OD values between sero-positive hospital cases, outpatient cases and controls. Alternatively, older people may be at particular risk of scrub typhus. Older people may be more exposed to infectious mite larvae due to behavioural factors, which in our view is not very likely. They may also be at higher risk of infection compared to younger people when exposed to infectious mite larvae because of age-related changes in skin anatomy, physiology and immunology.

The ELISA tests used in this study contain the recombinant antigens of Karp, Kato, Gilliam and TA716. Due to the great antigenic diversity of Orientia tsutsugamushi strains [28] these diagnostic assays may not detect scrub typhus-specific antibodies of all strains. In a study from South India, 16 out of 240 nested PCR confirmed scrub typhus cases were negative for serological scrub typhus tests [19]. These 16 samples were taken predominantly in the first seven days of illness during which serological tests may often be negative. These findings suggest that non-recognition of antibodies by the IgG ELISA tests applied to convalescent samples in the present study is unlikely to strongly affect estimates.

While the enrolment of cases and controls in villages may approximately have represented a near-random selection process, the enrolment of villages did not follow a random or equivalent systematic sampling procedure. The study aim was to identify endemic villages and approximately measure the incidence in these for the purposes of informing the design of a larger study. It may be inappropriate to refer to this study as population-based in the strict sense. But even if villages had been selected at random, the study highlights the difficulties in defining a suitable target population for inference when studying infections occurring in distinct foci. As done in this analysis, the most suitable approach may be to define the target population based on quite easily obtainable sero-prevalence data, an approach that has been found useful to classify areas with respect to transmission intensity of dengue fever [29]. We therefore believe that our results, when stratified by sero-prevalence, can be generalised to other villages in South India with similar sero-prevalence levels. Whether or not the association between sero-prevalence and scrub typhus incidence applies to other scrub typhus endemic areas in Asia needs to be confirmed. The high burden of scrub typhus generally observed in Vellore district may indicate a particularly high risk in this area or greater awareness of the infection and better availability of scrub typhus diagnostics. In hospital-based studies conducted in other parts of India such as Puducherry, Goa and Chandigarh, scrub typhus was responsible for 24% to 41% of cases with undifferentiated fever [30–32]. These findings suggest that scrub typhus risk our study area may not be unusually high. This calls for sero-prevalence studies and perhaps similar low-cost nested case-control studies as this one in the wider region to understand the burden of scrub typhus. Being one of the first attempts to measure the incidence of scrub typhus in the community not relying on passive case finding, we believe the results despite their limitations give an approximate estimate of the true burden of scrub typhus in endemic settings. Future studies using a similar retrospective design should employ a shorter recall period (e.g. a second survey round in the middle of the season) and thorough training and supervision of field staff to reduce recall error. Our findings can more reliably be confirmed by conducting an adequately sized prospective cohort study, which we think is feasible. There seems little doubt that scrub typhus is an important infection from the public health perspective, deserving the allocation of larger research funds than are currently made available.

## Supporting information

S1 Table STROBE Statement—Checklist of items that should be included in reports of observational studies. (DOCX)

S1 Fig Optical densities (IgG) for controls, outpatient cases and hospital cases. (TIF)
