# Supplementary figures and images for: Hospitalisations and outpatient visits for undifferentiated fever attributable to scrub typhus in rural South India: Retrospective cohort and nested case-control study
Source: PLoS Negl Trop Dis. 2019 Feb 25;13(2):e0007160. doi: 10.1371/journal.pntd.0007160 (PMC6405239; doi:10.1371/journal.pntd.0007160)

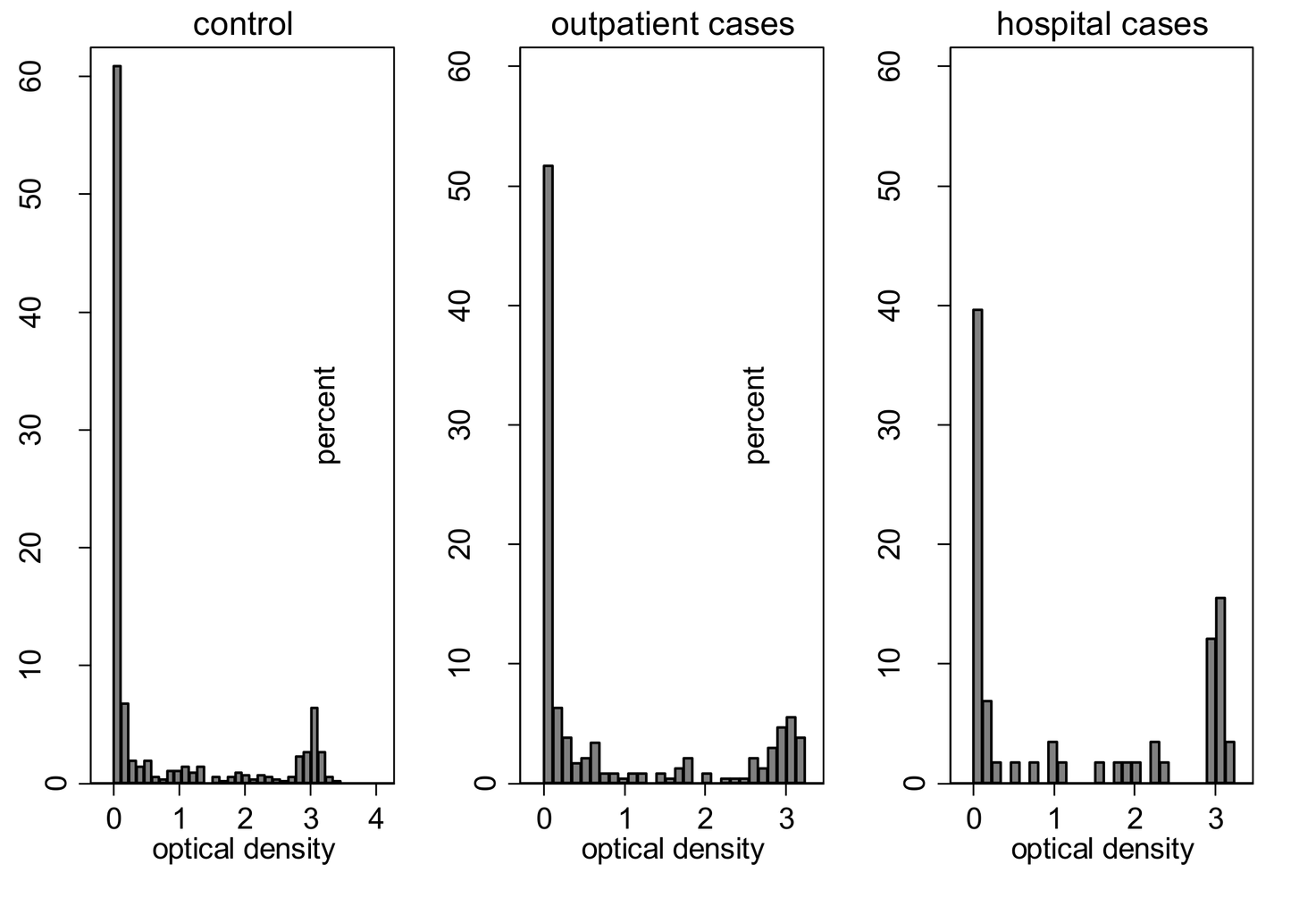

Supplement: S1 Fig — (TIF) [file pntd.0007160.s002.tif]
